# Errors in Recall of Age at First Sex

**DOI:** 10.1371/journal.pone.0072947

**Published:** 2013-08-28

**Authors:** Wenbin Liang, Tanya Chikritzhs

**Affiliations:** National Drug Research Institute, Curtin University, Perth, Western Australia, Australia; Tulane University, United States of America

## Abstract

**Aims:**

To measure the degree and direction of errors in recall of age at first sex.

**Method:**

Participants were initially recruited in 1994–1995 (Wave I) with 3 subsequent follow-ups in: 1996 (Wave II); 2001– 2002 (Wave III); and 2007–2008 (Wave IV). Participants' individual errors in recall of their age at first sex at Wave IV were estimated by the paired difference between responses given for age at first sex in Wave I and Wave IV (recalled age at first sex obtained at Wave IV minus the age at first sex obtained at Wave I).

**Results:**

The mean of the recall-estimation of age at first sex at Wave IV was found to be slightly increased comparing to the age at first sex at Wave I (less than 1 year). The errors in the recalled age at first sex tended to increase in participants who had their first sex younger or older than the average, and the recalled age at first sex tended to bias towards the mean (i.e. participants who had first sex younger than the average were more likely to recall an age at first sex that was older than the age, and vice versa).

**Conclusions:**

In this U.S. population-based sample, the average recall error for age at first sex was small. However, the accuracy of recalled information varied significantly among subgroup populations.

## Introduction

Age at first sex (vaginal intercourse) is a strong predictor of the risk of Sexually Transmitted Diseases (STD)s as well as STD related comorbidities [1], [2], [3]. Because of the strong relationship between age at first sex and risk of STDs, history of age at first sex is often collected and adjusted for in epidemiological studies on STDs and related comorbidities [3], [4], [5], [6], [7]. Recalled age at first sex is often used as a proxy measure of the age at first sex, especially in cross-sectional studies and case-control studies [3], [5], [7], [8], [9]. Attempts at estimation of events by recalled information after a long period of time are subject to bias due to memory error and could further affect the validity of epidemiological studies that rely on recalled information [10], [11]. Degree and direction of specific types of recalled measures have been investigated. For example, the reliability and validity of recall information on the age at first use of tobacco and alcohol have been examined in a number of studies [12], [13], [14], [15]. However, there is very limited data on the accuracy of recalled age at first sex. The National Longitudinal Study of Adolescent Health (Add Health) is a longitudinal study with a representative U.S. sample of adolescents in grades 7-12 at baseline. Data collected from the Add Health survey have been used previously to investigate the reporting of sexual activities among adolescents and young adults [16], [17]. This study aims to measure the degree and direction of errors in recall of age at first sex using data collected from the Add Health survey.

## Methods

Details of the National Longitudinal Study of Adolescent Health (Add Health) have been described in detail previously [18]. Public-use data collected from Waves I and IV of the Add Health study were used in this study. Participants were initially recruited in 1994–1995 (Wave I, mean age 15.75 years) with 3 subsequent follow-ups in: 1996 (Wave II, mean age 16.02 years); 2001– 2002 (Wave III, mean age 22.16 years); and 2007–2008 (Wave IV, mean age 28.67 years). A combination of self-administered questionnaires and interviews were employed to collect social, psychological and health information, including demographics, risk behaviors, health status and family composition. Information on age at first sex in both Wave I and Wave IV were collected during interview. Given the sensitive nature of the topic, in Wave I, data was collected using ACASI (Audio Computer Assisted Self Interview) while an interviewer was present during the process. Participant's age at first sex estimated at Wave I was calculated by subtracting the calendar year and month at first sex from the calendar year and month at birth. In Wave I, participants were asked “In what [month and] year did you have sexual intercourse for the very first time?” then at Wave IV (over 10 years later), participants were asked “How old were you the first time you ever had vaginal intercourse?” In order to ensure that age at first sex obtained from Wave I represented the age at first sex, this study included participants who were 18 years or younger at Wave I and who reported the age at first sex (virginal intercourse) in Wave I and in Wave IV.

### Data analysis

Participants' individual errors in recall of their age at first sex at Wave IV were estimated by the paired difference between responses given for age at first sex in Wave I and Wave IV (recalled age at first sex obtained at Wave IV minus the age at first sex recalled at Wave I). Age at first sex recalled at Wave I was used as a proxy of actual age at first sex. In the descriptive analysis, the mean recall error was estimated for the whole sample and repeated for each gender and each quartile of age at first sex estimated from Wave I. In the multivariate analysis, recall error was divided into three groups by its 25th percentile (−0.92) and 75th percentile (1.00). This categorical variable was then used as the dependent variable in the multinomial logistic regression model to investigate the effects of demographics, socioeconomic, general health and sexual behaviors. Demographic variables included age at wave IV, gender and race. Highest educational level and health insurance cover were included in the model as a proxy measure for socioeconomic status. Self-reported health status was used as a measure of general health status. In order to account for variation in sexual behaviors, sexuality of participants and total number of sex partners were also included in the model. Sampling weight supplied by the Add Health study was also applied in all analyses.

## Results

In Wave I, there were 1,774 participants aged 18 years or younger who reported age at first sex within the public use data file. Some 75% (1,337) were successfully followed-up at Wave IV, having provided recalled information on age at first sex, and they were therefore included in this study. Overall, the mean age at first sex obtained from Wave I was 14.38 (95% CI 14.24 – 14.52); the mean age at first sex obtained from Wave IV was 14.74 (95% CI 14.62 – 14.85); and the mean of the paired difference was 0.36 (95% CI 0.20 – 0.51). This indicates that on average, age at first sex recalled at Wave IV was slightly but significantly older than age at first sex estimated at Wave I. When the mean of the paired difference was estimated by gender and the age at first sex (categorized into quartiles), the largest discrepancies were found among participants who had first sex at the earliest age (in the first quartile) such that they over-estimated age at first sex by an average of 2.96 years for males and 1.80 for females. Participants in the 4th quartile for age at first sex showed the largest negative values indicating that their ages at first sex recalled at Wave IV tended to be younger than their age at first sex estimated at Wave I (Table 1).

**Table 1:** Recall errors by gender and quartiles of actual age at first sex^1^.

	Male			Female		
	Mean (years)	95%	CI	Mean (years)	95%	CI
Age at first sex estimated on Wave I						
1^st^ quartile [0, 13.6)^2^	**2.96**	2.47	3.45	**1.80**	1.07	2.53
2^nd^ quartile [13.6, 14.9)	**0.08**	−0.32	0.47	−**0.20**	−0.40	0.00
3^rd^ quartile[14.9, 16)	−**0.18**	−0.47	0.11	−**0.35**	−0.50	−0.20
4^th^ quartile (16, 18]	−**0.94**	−1.23	−0.64	−**0.76**	−0.99	−0.52

1 Recall errors were calculated by subtracting age at first sex recalled at Wave I from age at first sex recalled at Wave IV.

2 [ ] and ( ) were used as notations for closed and opened intervals, respectively.

Similar associations were found between the recall errors and age at first sex in the multivariate analysis. Among participants who had first sex before 13.6 years (25 percentile), the age at first sex recalled at Wave IV was significantly more likely to be older than the age at first sex estimated at Wave I compared to participants who had their first sex between 14.9 years (50 percentile) and 16 years (75 percentile). Conversely, the age at first sex recalled at wave IV were more likely to be younger than the age at first sex estimated at wave I among participants who first had sex after 16 years. In addition, multivariate analysis showed that females were significantly less likely to recall an age at first sex that was one year or older than the actual age. Participants who had achieved the highest education level were more likely to provide a more accurate recalled age at first sex, and were significantly less likely to recall an age at first sex that was 0.92 years younger than the age at first sex estimated at Wave I (Table 2). It was also observed that the total number of sexual partners reported by participants was inversely associated with the difference between the ages at first sex obtained in Wave I and Wave IV (p for trend <0.01).

**Table 2:** Recall error variation by participant characteristics.

	First quartile of recall error			Last quartile of recall error		
	RRR^1^	95% CI		RRR	95% CI	
Sex						
male	1.00			1.00		
female	0.80	0.54	1.17	0.32	0.20	0.52
Race						
white	1.00			1.00		
black	1.44	0.99	2.11	2.98	1.98	4.47
other	0.74	0.31	1.81	1.07	0.39	2.91
Age at Wave IV (in years)						
1^st^ quartile [25.2, 28.5)	0.88	0.50	1.54	0.96	0.54	1.72
2^nd^ quartile [28.5, 29.5)	0.82	0.50	1.32	1.15	0.61	2.15
3^rd^ quartile [29.5, 30.3)	1.00			1.00		
4^th^ quartile [30.3, 31.5]	1.10	0.71	1.71	1.35	0.75	2.42
Age at first sex (in years)						
1^st^ quartile [0, 13.6)^2^	0.57	0.30	1.11	10.92	6.17	19.33
2^nd^ quartile [13.6, 14.9)	0.85	0.53	1.37	2.22	1.29	3.81
3^rd^ quartile [14.9, 16)	1.00			1.00		
4^th^ quartile [16], [18]	1.68	1.07	2.65	0.37	0.14	0.99
Health						
excellent, very good or good	1.00			1.00		
fair or poor	1.03	0.62	1.71	0.92	0.50	1.68
Highest education achieved						
didn't finish high school or lower	1.00			1.00		
high school graduate	0.55	0.30	1.00	0.96	0.51	1.78
with some college education or above	0.26	0.14	0.48	0.57	0.31	1.05
Health insurance in the last 12 months						
without cover	1.00			1.00		
with cover but less than 12 months	1.04	0.59	1.84	0.82	0.45	1.48
with cover for 12 months	1.49	0.93	2.39	0.73	0.44	1.20
Sexuality						
100% heterosexual	1.00			1.00		
other	0.89	0.54	1.47	1.01	0.54	1.90
Total number of sex partners over lifetime						
1 to 5	0.85	0.48	1.51	1.56	0.80	3.05
6 to 9	1.00			1.00		
10 to 19	1.18	0.70	2.00	0.64	0.34	1.20
20 or more	1.67	0.99	2.80	0.49	0.26	0.92

1 Relative-risk ratio

2[ ] and ( ) were used as notations for closed and opened intervals, respectively.

* −0.92 and 1 were the 25 and 75 percentile, respectively.

* Recall error falling within the 25−75 percentile were used as the base-level in the multinomial logistic regression (in a similar manner to the control group in a logistic regression)

## Discussion

In this longitudinal study, information on age at first sex collected before the age of 18 at Wave I was compared to the recalled age at first sex which was collected 12 years+ later at Wave IV. There was very limit data available in the published literature measuring the error of recalled age at first sex. The mean of the recall-estimation of age at first sex from Wave IV was found to be slightly increased comparing to the age at first sex estimated at Wave I. This finding was similar to the observations from studies which investigated the reliability of recalled information on first use of tobacco. Recalled age at first use of tobacco has been showed to increase slightly overtime [12], [13], [14], [15]. Since the mean difference was small (less than 1 year), this bias would likely have only have minimal effects on surveys which aim to estimate the population average age at first sex.

Nevertheless the errors in the recalled age at first sex tended to increase among participants who had their first sex younger or older than the average, and the recalled age at first sex tended to bias towards the mean (i.e. participants who had first sex younger than the average were more likely to recall an age at first sex that was older than the actual age, and vice versa). This suggests that retrospectively collected information on age at first sex in adulthood may omit a certain degree of individual variability. This may have led to the recalled age at first sex being more similar (i.e. less variation) compared to actual age at first sex. Using recalled information of age at first sex may reduce the power of analysis when investigating the effect of age at first sex on a given outcome; or increase the residual confounding effects of age at first sex, when age at first sex is considered as a potential confounding factor, and is controlled for in analyses.

In addition, this study also showed that the size and direction of recall errors was significantly associated with demographic variables including: gender; race; highest educational level achieved (a proxy measure of socio-economic status); and, total number of sex partners. Gender, race and highest education level are commonly controlled for in epidemiological studies that investigate the effect of age at first sex; these adjustment approaches may off-set the potential impact of recall bias (i.e. due to their correlation with recall error). Future retrospective studies which require recalled information on age at first sex may benefit from improving the accuracy of the recalled information. Digital media may present one option for improving the accuracy of recalled information. Digital photos, videos, messages, blogs and electronic calendar data, have been widely used to capture and share day-to-day life. For many individuals, these may in effect constitute a digital library of their past experiences and drawing upon such resources may facilitate more accurate retrieval of personal historical events including age at first sex [16].

This study is subject to some limitations. Firstly it should be noted that slight difference in the interview process between the two surveys may have contributed to a small proportion of recall errors. Secondly, participants at younger ages may have been more likely to misinterpret the concept of “sexual intercourse” which was intended to refer to vaginal intercourse but may have been more broadly interpreted by some to include oral sex only encounters. In addition, this study was based on a representative U.S. sample and future investigations repeated among other populations will be needed to demonstrate generalizability of the findings.

## Conclusion

In this U.S. population-based sample, the average recall error for age at first sex was small. However, the accuracy of recalled information varied significantly among subgroup populations.
